# Editorial: Advances in the understanding and management of non-tuberculous mycobacteria infections

**DOI:** 10.3389/ftubr.2026.2012273

**Published:** 2026-10-20

**Authors:** Anil K. Ojha, Laurent Kremer

**Affiliations:** 1 Wadsworth Center, New York State Department of Health, Albany, NY, United States; 2 Department of Biomedical Sciences, University at Albany, Albany, NY, United States; 3 Centre National de la Recherche Scientifique UMR 9004, Institut de Recherche en Infectiologie de Montpellier (IRIM), Université de Montpellier, Montpellier, France; 4 INSERM, IRIM, Montpellier, France

**Keywords:** epidemiology, infection, nontuberculous mycobacteria, pathogenesis, physiology, treatment

Nontuberculous mycobacteria (NTM) infections are increasingly recognized as an important and complex challenge in respiratory medicine. Unlike *Mycobacterium tuberculosis*, NTM are ubiquitous environmental organisms, yet several species can cause chronic pulmonary disease (PD) that is difficult to diagnose, difficult to eradicate, and associated with substantial morbidity. The challenge is particularly evident with *Mycobacterium abscessus*, an organism characterized by remarkable physiological adaptability and limited therapeutic options (1). The contributions assembled in this Research Topic, “*Advances in the Understanding and Management of Non-Tuberculous Mycobacteria Infections*,” provide complementary perspectives on this problem, spanning epidemiology, diagnosis, bacterial physiology, host immunity, and therapeutic innovation. Most of the contributions were presented at the 2025 edition of the Colorado Mycobacteria conference: Nontuberculous mycobacteria, thereby representing the theme of the conference and the future direction of NTM research.

The central message emerging from these studies is that NTM-PD cannot be understood through any single lens. Its clinical complexity reflects the interaction between the epidemiological context in which exposure occurs, the biological diversity of the organism, the anatomical and immunological environment of the infected lung, and the pharmacological limitations of available treatments. Therefore, progress will depend on connecting these areas rather than considering them as independent challenges.

The epidemiological contribution by Baham et al*.* illustrates one of the first obstacles: defining the burden of NTM disease. Analysis of eight years of hospital discharge data in Texas identified more than 12,000 NTM diagnosis-coded inpatient discharges over 23 million records, particularly affecting elderly Caucasian women with substantial geographical variation and considerable mortality among affected patients. The findings of this study highlight the difficulty of accurately measuring a disease whose diagnosis requires integration of clinical, radiological, and microbiological evidence.

Better epidemiological information must be accompanied by better diagnostics. The review by Singh et al. highlights the continuing difficulty of distinguishing NTM infection from colonization and of differentiating NTM-PD from other chronic respiratory infections, including tuberculosis. Species-level identification is particularly important because pathogenic potential and antimicrobial susceptibility vary substantially among NTM species. Advances in molecular diagnostics, MALDI-TOF, whole-genome sequencing, artificial intelligence and luciferase reporter mycobacteriophage (LRM)-based assays offer opportunities to accelerate identification and improve characterization of resistance. The fundamental diagnostic question remains whether detection of an organism represents clinically meaningful disease.

The biological studies in this volume provide important insight into why *M. abscessus* can be so difficult to eradicate. Canestrari et al. investigate the SigM regulatory network and characterize a regulon that includes ESX-4 secretion system, implicated in pathogenesis of *M. abscessus* (2)*.* Their study further emphasizes how the relationship between secretion systems and stress-responsive transcription regulation pathways are integral to the physiology and pathogenic potential of this organism. Such work is important because it shifts attention from individual resistance genes toward complex interconnected regulatory systems that allow bacteria to adapt to hostile environments.

Amin et al*.* extend this concept by examining the accessory genome of *M. abscessus*. They identify the VapB5-VapC5 toxin-antitoxin system as a determinant that reshapes bacterial physiology during antimicrobial stress. VapC5 induces growth arrest, modulates the WhiB7 regulatory program, and promotes a persistent physiological state. These findings establish VapC5 as a broad physiological modulator that selectively reshapes *M. abscessus* antimicrobial susceptibility based on drug mechanism. These findings suggest that bacterial genotype may ultimately inform not only whether an isolate is resistant, but also which antimicrobial mechanisms are most likely to remain effective.

The host environment adds another level of complexity. Haist et al. demonstrate that the anatomical site of *M. abscessus* infection influences the immune response and the contribution of individual macrophage populations. In their murine models, infection involving obstructed airways produced a different cellular environment from alveolar infection, and resident alveolar macrophages contributed differently to bacterial control depending on the site of infection. These findings are particularly relevant to patients with structural lung disease, in whom abnormal airways may create localized environments that favor persistence despite substantial immune activation.

Overall, these bacterial and host studies suggest that persistence should not be viewed simply as an intrinsic property of the microorganism. It is an emergent phenomenon arising from the bacterial adaptative response to the environment in which the infection occurs. This perspective may be particularly important for *M. abscessus*, where prolonged antimicrobial therapy frequently fails to achieve durable microbiological clearance. Therapeutic innovation therefore needs to address both bacterial physiology and the limitations of drug exposure. Park et al. explore this principle using physiologically based pharmacokinetic modeling of inhaled tigecycline. Rather than increasing systemic exposure to achieve greater pulmonary concentrations, this approach seeks to deliver the drug directly to the lung while limiting systemic exposure and associated adverse effects. The work underscores the importance of pharmacological optimization.

This Research Topic provides a coherent picture of a field moving toward a greater clarity on prevalence and pathogenesis of *M. abscessus* infections to achieve effective therapy. Epidemiological studies can define where the burden lies; advanced diagnostics can determine which organisms are present and help characterize their resistance potential; studies of bacterial physiology can reveal mechanisms of persistence; investigation of host responses can explain why infection behaves differently in distinct pulmonary niches; and pharmacological modeling can identify strategies for improving drug exposure while reducing toxicity. The next step is to connect these advances. The future management of NTM-PD is unlikely to be based on a universal regimen applied to every patient with the same species. Instead, treatment may increasingly depend on a combination of species and strain characteristics, molecular resistance determinants, bacterial physiological state, anatomical distribution of infection, host factors, and drug penetration into the relevant pulmonary compartment. Achieving this goal will require prospective clinical studies capable of validating biomarkers and molecular predictors identified in experimental systems, as well as standardized approaches that allow findings from epidemiology, microbiology, immunology, and pharmacology to inform one another.

## Concluding remarks

The Research Topic therefore highlights both how far the field has progressed and how much remains unresolved. We now have increasingly sophisticated tools to detect NTM, characterize their genomes and regulatory networks, investigate host responses, and model innovative therapeutic approaches. The challenge will consist to transform these advances into clinically validated strategies that improve outcomes for NTM patients. Ultimately, the management of NTM infection will require moving beyond the concept of simply finding and killing a difficult bacterium. We need to understand where and how these microorganisms persist, how they adapt among their different hosts, why the host fails to easily clear them, and how therapy can be delivered effectively to the site of disease.
